# Advances in the Electrochemical Detection of Antibiotics: Modified Materials, Wearable Sensors, and Future Prospects

**DOI:** 10.3390/s25175541

**Published:** 2025-09-05

**Authors:** Xun Gong, Yingying Li, Xin Li, Jie Hu, Xin Zhou, Xiupei Yang

**Affiliations:** 1 College of Chemistry and Chemical Engineering, China West Normal University, Nanchong 637000, China; gx_2333@163.com (X.G.); 17883357270@163.com (Y.L.); lx609638229@163.com (X.L.); 15828402449@163.com (J.H.); zxxxn2020@163.com (X.Z.); 2 Precise Synthesis and Function Development Key Laboratory of Sichuan Province, Nanchong 637000, China

**Keywords:** electrochemical sensors, modified materials, antibiotics, wearable sensors

## Abstract

**Highlights:**

This review highlights advances in electrochemical sensors for antibiotic detection, focusing on innovative materials like MOFs, COFs and quantum dots that enhance sensitivity and detection limits. It discusses wearable sensors for real-time clinical and food safety monitoring via smartphone integration, and emphasizes nanocomposite synthesis and sensor miniaturization for rapid, low-cost antibiotic residue analysis.

**What are the main findings?**

- Advanced materials (MOFs, COFs, quantum dots) enhance electrochemical sensors’ sensitivity, selectivity, and detection limits for antibiotics.
- Wearable sensors enable real-time antibiotic monitoring in clinical/food safety via smartphone integration.

**What is the implication of the main finding?**

- Enhanced materials improve rapid, low-cost detection of antibiotic residues in environment/food.
- Wearable/smartphone-integrated sensors revolutionize on-site monitoring, reducing human exposure risks.

**Abstract:**

Antibiotics, valued for their remarkable efficacy, are widely employed across diverse domains. However, their rampant overuse has precipitated severe environmental and health crises, necessitating the development of efficient techniques for rapid and selective antibiotic detection. Electrochemical detection has emerged as a highly promising approach, offering unmatched advantages such as cost-effectiveness, speed, and reliability. The field has witnessed significant advancements through the innovation of advanced electrode modification materials. This review provides a comprehensive analysis of recent progress in the development and application of modified materials for antibiotic detection. Furthermore, the increasing need for real-time monitoring has spurred the development of wearable electrochemical sensors, which are revolutionizing applications in human health and food safety. Looking ahead, future research is poised to focus on synthesizing nanocomposites with superior electrochemical properties and advancing the miniaturization of sensors, promising transformative practical applications in antibiotic detection.

## 1. Introduction

Antimicrobial agents, commonly referred to as antibiotics, represent a category of chemical compounds capable of exerting targeted suppressive effects on specific organisms even at minimal concentrations. These biologically active substances are primarily synthesized as secondary metabolic products by microorganisms, though they may also originate from higher plants and animals, exhibiting properties that can disrupt the cellular functions of pathogenic organisms [1]. Fundamentally, these compounds regulate microbial proliferation by targeting critical bacterial processes, including inhibition of cellular wall biosynthesis, modification of membrane permeability, disruption of protein production, and suppression of genetic material replication and expression. In clinical practice, antibiotics serve as indispensable therapeutic agents in combating bacterial infections, playing a pivotal role in modern healthcare. Beyond human medicine, these compounds are widely utilized in veterinary practices for the prevention and management of diseases in livestock, thereby supporting the sustainable growth of the animal husbandry sector. The extensive application of these pharmacological agents has significantly enhanced public health outcomes and advanced the productivity of the agricultural and aquaculture industries [2].

Tens of thousands of antibiotics have been discovered that can be obtained via microbial metabolism and synthetic and artificial semisynthetic routes. Semisynthetic antibiotics are various derivatives of antibiotics obtained by biosynthesis via chemical, biological or biochemical methods to modify their molecular structure. In addition, classified according to their chemical structure, antibiotics exhibit different antibacterial activities on the basis of the different functional groups in their respective chemical structures and can be categorized into quinolone antibiotics and β-lactam antibiotics [3,4]. The classification of common antibiotics is shown in Table 1.

The problems of environmental pollution and food contamination caused by antibiotic misuse are becoming increasingly serious. However, in recent years, owing to the large and frequent use of antibiotics, the problem of antibiotic pollution in water, including surface water [5,6], groundwater, drinking water [7], sewage [8] and aquaculture wastewater, has become quite common. Among them, the detected concentration level in wastewater is μg·L^−1^ to mg·L^−1^, which is a trace pollution level, whereas the detected concentration level in other water bodies is ng·L^−1^, which is a trace pollution level. As our environment becomes contaminated with antibiotics, an increasing number of bacteria are exposed to low concentrations of antibiotics, increasing antibiotic resistance. Studies [9,10,11] have shown that antibiotics have a variety of adverse effects on humans, such as direct toxic effects, allergic reactions, potential organ toxicity, teratogenicity, and carcinogenicity, as well as causing the emergence of many antibiotic-resistant bacteria in organisms or in the environment, thus increasing the difficulty of the clinical use of medication and threatening the safety of human life. The misuse of antibiotics can lead to excess residues in edible meat, offal or metabolic byproducts of animals. These results show that the problem of antibiotic pollution has become prominent, the pollution situation is serious, and its environmental risks and ecotoxicological effects cannot be ignored.

Currently, a variety of instruments and methods are used to detect antibiotics. Chromatography [12,13], spectrophotometry [14,15] and capillary electrophoresis [16] are classical methods for detecting antibiotics. For instance, Xu et al. combined liquid chromatography–tandem mass spectrometry and adopted a simple one-step purification process, which could simultaneously determine 55 kinds of antibiotics in aquatic products [17]. The enzyme-linked immunosorbent assay (ELISA) [18] is also a commonly used method and is based on the principle of using the specific binding of antibodies to antibiotics for detection. Although the above techniques have high sensitivity and can achieve the simultaneous detection of multiple antibiotic residues, they have several disadvantages. High-performance liquid chromatography (HPLC) has a good effect but high cost. ELISA can be used to obtain direct results, but the results are prone to false positives, and spectrophotometry is simple but inaccurate. In recent years, electrochemical sensors have emerged in the field of antibiotic detection. Electrochemical sensors have unique advantages in that they are relatively inexpensive, easy to operate, and can produce rapid results. Moreover, it can be miniaturized and fluently combined with smartphones [13] in onsite or immediate detection scenarios, providing strong support for antibiotic residue monitoring in the environment and food.

## 2. The Basic Principle of Electrochemical Sensors

An electrochemical sensor is composed of an electrode device, an electrolyte and an electrochemical workstation. Specifically, the electrode device includes a working electrode, reference electrode and counter electrode. An electrochemical sensing signal, such as current, potential, or conductance, is generated during electrochemical sensing and is usually proportional to the analyte concentration [19]. The generation of these signals is closely related to the redox reactions occurring on the surface of the working electrode in the electrochemical sensing system. When the target analyte undergoes redox on the surface of the modified electrode, it triggers an electron transfer process, which leads to a change in current or potential, thus providing a basis for qualitative and quantitative detection of the analyte.

Electrochemical sensing methods can be categorized into two types: direct detection and indirect detection. The direct detection mechanism means that the target analyte is directly involved in the electrochemical reaction on the electrode surface and can produce a detectable electrochemical signal. Some antibiotics, such as metronidazole, nitrofuranone and chloramphenicol, contain active nitro groups that support redox reactions and can direct redox signals in electrochemical assays. This direct detection mechanism has the advantages of simple operation and is effective for the detection of electrochemically active substances. However, for antibiotics that are difficult to use directly in redox reactions, indirect assays can often work. In the indirect detection mechanism, one or more intermediary substances are introduced. These intermediates are able to react or interact specifically with the target antibiotics and have high electrochemical activity. Alternatively, various specific target recognition pairs can be modified on the working electrode material. When the target drug is identified, the resistance value of the electrode material on the surface of the working electrode will be affected, thus affecting the electrochemical signal [20]. Indirect detection broadens the application of electrochemical sensing and enables the detection of a wider range of antibiotics, but the detection system is more complex than direct detection is because of the involvement of multiple reaction steps and interactions between substances.

Electrochemical analysis relies on measurable electrical signals generated by electrochemical sensors, which can be voltammetry, potentiometry, conductivity or impedance. Typically, the electrical signal is positively correlated with the concentration of the analyte, reflecting not only the content of the target antibiotic but also the ability of the modified material to transfer electrons. Electrical signals can be detected by various methods, such as cyclic voltammetry (CV) [21,22], electrochemical impedance spectroscopy (EIS) [23,24], differential pulse voltammetry (DPV) [25], square wave voltammetry (SWV) [26], stripping voltammetry (SV) [27], linear sweep voltammetry (LSV) and amperometry (i-t) [28].

Cyclic voltammetry can extract a large amount of useful information, such as the reversibility of the reaction, the number of transferred electrons, and the role of diffusion in the solution or the pre-adsorption of analytes on the electrode surface [29]. CV can also be used for quantitative analysis to determine the concentration of a specific analyte based on the magnitude of the oxidation peak current. When only forward scanning is included, this technique is called linear scanning voltammetry (LSV). EIS can provide information about the kinetics of electrode processes and the interface structure, which is suitable for studying electrode reaction mechanisms. Compared with CV, differential pulse voltammetry (DPV) measures a differential current, which can reduce the influence of charging current [30]. DPV can enhance the analysis of Faraday current, making it one of the most sensitive electrochemical methods [29]. With good sensitivity, DPV is especially suitable for the quantitative analysis of trace levels. Similar to DPV, SWV can also be used for quantitative detection [31], but is faster than DPV. In addition, the current difference in the SWV method can provide more information about reversible reactions than DPV by recording the forward and reverse currents [32]. In addition, stripping voltammetry (SV) [27] and linear sweep voltammetry (LSV) [33] are useful in special situations. SV method enriches the substance to be tested through pre-electrolysis and then dissolves it in reverse. It can achieve quantitative analysis of trace substances and has a relatively high sensitivity. The characteristic peaks of LSV are related to the reversibility of the reaction and are often used in the study of electrode reaction mechanisms. Amperometry (i-t) is the measurement of Faraday currents generated by the oxidation/reduction of electroactive analytes. Such changes in current response can occur rapidly within seconds [29].

## 3. Materials for the Construction of Antibiotic Electrochemical Sensors

The properties of modified materials in electrochemical sensors are crucial for their analytical performance. Common modifying materials for the preparation of sensor interfaces include carbon nanomaterials [34], metal and metal oxide nanomaterials [35,36], and molecular imprinted polymers (MIPs) [37], which have large surface areas, excellent electrical conductivity, highly efficient electrocatalytic activity, and good biocompatibility. These properties are closely related to factors such as the composition, morphology and structure of the nanomaterials. Table 2 lists a range of commonly used modifying materials and their applications in different sample matrices.

### 3.1. Carbon Nanomaterials

Carbon nanomaterials have many advantages, including easy preparation, low ohmic resistivity and robustness. Additionally, carbon materials possess a large specific surface area, providing abundant active sites that form the foundation for enhancing detection signals. Researchers have confirmed that carbon nanomaterials can further expand the effective active surface area of electrodes, enhancing their conductivity and stability [38,39,40]. In addition, they have been widely used as support substrates and effectively combined with active substances to improve their electrocatalytic behavior. Carbon materials also have good biocompatibility and have been widely used in the field of bioelectrochemical detection. The biocompatibility and biodegradability of carbon nanomaterials make them ideal active materials for wearable devices [41]. The following will introduce the main applications of carbon nanotubes and graphene.

**Table 2:** Performances of electrochemical sensors based on different materials.

Modifier	Electrode	Technique	Liner Range (µM)	LOD (µM)	Analytes	Sample	Ref.
Carbon nanomaterials	Z-800/rGO/GCE	DPV	1~180	0.25	Chloramphenicol	Milk, honey	[42]
	Perlberg/GCE	DPV	5~225	2.2	Tetracycline	Urine	[43]
	Fe_3_O_4_/MWCNT/CPE	DPV	0.3~100	0.09	Enrofloxacin	Milk, egg, honey, chicken	[44]
	CDs-Ag@Cu2O-GA/GCE	DPV	10~110	0.71	Metronidazole	Milk	[45]
	3DCNTs@CuNPs@MIP	CV	10~500	10	Chloramphenicol	Milk	[46]
	VS_2_/Ti_3_C_2_T_x_ MXene-SPCE	LSV	0.01~400	0.0047	Nitrofurantoin	Lake, milk, honey	[47]
	GO-MXene-PDDA/SPCE	CV	0.04~200	0.00122	Furazolidone	Piped water, river water	[48]
	OMC@Ti_3_C_2_ MXene/Apt/SPCE	DPV	0.010~2	0.00351	Kanamycin	Aminoglycoside antibiotics	[49]
Metal and metal components nanomaterials	SbFE/GCE	SWASV	0.40~3.00	0.15	Tetracycline	Honey	[50]
	Ni/MoN/MCGCE	DPV	5~150	0.008	Tinidazole	Tablet	[51]
	TCN@Au NPs/GCE	EIS	0.5~3	0.2	Amoxicillin	Wastewater	[52]
	AuNPs/PdNPs/ErGO/GCE	SWV	30~350	9	Lomefloxacin and amoxicillin	Milk	[36]
	LaFeO_3_/rGO/GCE	DPV	0.2~1221	0.048	Metronidazole	Urine, milk	[53]
Molecular imprinted polymers	Hydrophilic MIPs-GMA/GCE	CV	0.028~2.8	2.2 × 10^−3^	Tetracycline	Egg	[54]
	MWCNTs@MIP/CKM-3/P-r-GO/GCE	DPV	5.0 × 10^−3^~4.0	1.0 × 10^−4^	Chloramphenicol	Milk, honey	[55]
	EMIP/GNU/GO/GCE	DPV	20.0~950	7.1	Cefixime	Serum, urine	[56]
	Ery-MIP/SPE	DPV	1.2 × 10^−4^ ~4.0 × 10^−4^	1 × 10^−4^	Erythromycin	Tap water	[37]
Covalent organic framework	AuNPs@COFs-MWCNTs/GCE	DPV	0.08~25	0.016	Doxorubicin	Human serum, cell lysate	[57]
	TAPB-PDA-COFs/AuNPs/GCE	SWV	0.05~10, 10~120	0.041	Enrofloxacin	Water, milk	[58]
	MIP/CuS/Au@COF/GCE	DPV	1.0 × 10^−5^ ~100	4.3 × 10^−6^	Sulfathiazole	Mutton, fodder	[59]
	MIP/MoS_2_/NH_2_-MWCNT@COF/GCE	DPV	0.30~200	0.11	Sulfamerazine	Pork, chicken	[60]
	COF@NH_2_-CNT/GCE	DPV	0.2~100	0.0775	Furazolidone	Beef, pork	[61]
Biofunctional materials	UiO-66-NH_2_@M^n+^/cDNA MB@Apt1-Apt2 capture probes	SWV	2 × 10^−6^~0.1	1.6 × 10^−7^	Kanamycin	Milk	[62]
	PEI/TetX2/NPGCE	CV	0.5~5	0.018	Tetracycline	Milk	[63]
	SPdCEs	I-t	1.92 × 10^−3^ ~0.454	3.9 × 10^−4^	Sulfapyridine	Milk	[64]
Quantum dots	QDs-P6LC-PEDOT:PSS/GCE	SWV	0.90~69.0	0.05	Amoxicillin	Milk, synthetic urine	[65]
	CdTe-CB-CTS: EPH/GCE	SWAdASV	0.2~7.4	6.6 × 10^−3^	Norfloxacin	Chitosan film	[66]
	Magnetic and aptamer-QDs EDP	SWV	3 × 10^−4^~0.93	9.3 × 10^−5^	Chloramphenicol	Fish	[67]

Carbon nanotubes (CNTs) can be categorized into single-walled carbon nanotubes and multiwalled carbon nanotubes, which are seamless and hollow tubular structures formed by carbon atoms as if they were curled from graphene. Due to the excellent electrocatalytic performance, high active surface area and outstanding mechanical and chemical properties of CNTs, they have aroused great interest in the field of electrochemistry [38]. Chemical doping by different molecules and the effect of charge transfer can affect their electrical characteristics [68].

**Figure 1:**
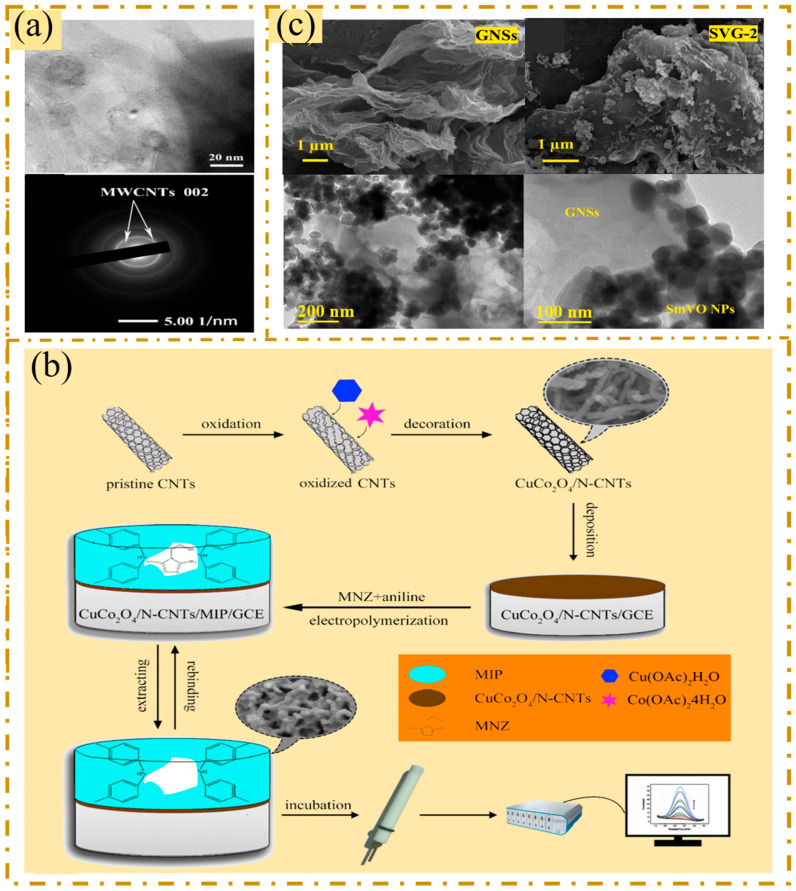
(**a**) High-resolution images and electron diffraction pattern of CNTs. Reproduced with permission from Ref. [69]. Copyright 2023, Elsevier. (**b**) Schematic diagram of the preparation of the CuCo_2_O_4_/N-CNT/MIP/GCE and the electrochemical determination of metronidazole. Reproduced with permission from Ref. [34]. Copyright 2019, Elsevier. (**c**) FESEM images of GNSs and as-prepared composites SVG-2, and HR-TEM images of SVG-2 at different magnifications. Reproduced with permission from Ref. [72]. Copyright 2022, Elsevier.

Using a low ligand–metal ratio controlled by organic ligands loaded on their surfaces, Wang et al. synthesized two-dimensional Ce-MOF nanosheet hybrid materials on the skeleton of carbon nanotubes [69]. The morphology of carbon nanotubes used is shown in Figure 1a. The Ce complex is uniformly chelated around the CNTs, forming a two-dimensional network composite layer with the CNTs as the supporting framework. The CNTs framework network enhances the conductivity and specific surface area of the composite layer, thereby improving the catalytic efficiency. Elfiky et al. utilized the synergistic effect between carbon materials by blending carbon paste (CP), flake graphite (FG) and multiwalled carbon nanotubes (MWCNTs) to make an electrochemical sensor for ofloxacin (OFX) [70]. The MWCNTs were functionalized with nitric and sulfuric acids to introduce functional groups such as hydroxyl and carboxyl groups, and the H^+^ ions on the surface easily exchanged cations with the positively charged piperazine group of OFX, resulting in strong adsorption of OFX. In addition, the interaction between the hydroxyl group of the MWCNTs and the fluorine group of OFX promoted adsorption. However, strongly adsorbed coatings may reduce conductivity, in which case crystalline flake graphite (FG) is added to reduce resistance. Crystalline flake graphite is a natural form of graphite and a promising electrode material for improving conductivity. Due to the synergistic effects of the two carbon materials (FG and MWCNTs), the developed electrochemical sensor exhibits significant electrocatalytic activity.

In addition, CNTs are easy to surface modify, and modified CNTs are conducive to fixation and have better biocompatibility [71]. As shown in Figure 1b, Wang et al. modified CuCo_2_O_4_ nanoparticles with nitrogen-doped carbon nanotubes (CuCo_2_O_4_/N-CNTs) to achieve a high specific surface area and good electrical conductivity [34]. Nitrogen doping increases the conductivity of carbon nanotubes and can enhance their stability. CuCo_2_O_4_/N-CNT improved the performance of the sensor by increasing the electrochemically active area and the electron transfer ability to achieve fast and sensitive detection of metronidazole.

Graphene has been widely employed in electrochemical sensors owing to its excellent properties, which include rich chemical reaction sites, a high surface-to-volume ratio, superior conductivity, rapid electron transfer ability, and catalytic performance [73,74,75,76]. Graphene nanosheets (GNSs), developed as two-dimensional materials, have many potential for use in electrochemical sensing. Babulal et al. combined samarium vanadate nanoparticles with graphene nanosheets (GNSs) by ultrasonication to obtain SmVO_4_-GNSs (SVG) nanocomposites [72]. The GNSs are in a stacked laminar structure with SmVO_4_ nanoparticles dispersed on their surface, as shown in Figure 1c. The 2D-fine layer of graphene nanosheets and the sphere-like shape of SmVO_4_ nanoparticles considerably increase the electron transfer by producing plentiful active areas in the electrochemical activity. It can be expected to enhance the electron transfer activity and the performance of more active sites. GNSs have a synergistic effect when combined with metal oxides, which enhances the electrochemical performance and stability of the sensor. The prepared sensor modified by SVG can significantly increase the reduction signal of furantoin with high sensitivity.

Further improvements in graphene, such as loading nanoparticles on the surface or introducing functional groups, can enhance the performance of graphene or achieve special detection effects [77]. Yi et al. prepared reduced graphene oxide (rGO) via a modified Hummur method and reduction and further introduced Pd nanoparticles (PdNPs) [78]. PdNPs were uniformly dispersed on the rGO surface, and the rGO/PdNP nanocomposites were formed by NH–Pd bonds between the PdNPs and the amine groups in the rGO. The rGO/PdNPs exhibited excellent electroactivity and high selectivity toward chloramphenicol. In addition, due to the large specific surface area and good biocompatibility of Graphene quantum dots (GQDs), it can be combined with aptamers for electrochemical sensing. Roushani et al. developed an electrochemical aptamer sensor based on GQDs for the highly sensitive detection of streptomycin [79]. GQDs functionalized with amine and thiol groups (GQDs-N-S) can bind different metal nanoparticles through -NH and -SH groups, which, in combination with an aptamer probe, make the sensor more stable and improve the conductivity. In addition, carbon black (CB) and ordered mesoporous carbon (OMC) have also been excavated and used as sensor modifiers for the detection of antibiotics.

### 3.2. Metal and Metal Component Nanomaterials

Metal nanoparticle-modified electrodes have advantages such as large specific surface area, fast electron and mass transfer rates, which can improve the analytical performance of sensors. The electrocatalytic performance of metal nanoparticles mainly depends on the particle size [80]. The smaller the dispersed particles are, the larger the surface area and the higher the electrical activity will be. Metal nanoparticles can be functionalized to prevent their aggregation and stabilize their dispersion. The metal nanoparticles commonly used to make sensors that detect antibiotics are Au, Ag, Pt, Cu, etc. [81,82,83]. Munawar et al. constructed 3D imprinted nanostructures in which copper nanoparticles (Cu NPs) were deposited on the surface of 3D carbon nanotubes (CNTs), which were then covered with molecularly imprinted polymers [50]. To deposit more Cu NPs, amino groups were introduced into the CNTs as reaction centers, and then, the Cu NPs were uniformly anchored on the surface of the CNTs. The deposition of Cu NPs on the surface of CNTs increases its surface roughness. Surface roughness is an important parameter that determines the processing efficiency of electrodes. Such a surface provides more binding sites for the capture of the analyte, thereby leading to its sensitive detection.

Gold nanoparticles (AuNPs) are widely used in electrochemical biosensors because of their biophilicity and superior electrical conductivity. He et al. used a portable thin-film gold electrode (TFGE) with AuNPs incorporated into the modifying material to increase the detection sensitivity [84]. The signal tags, the prepared gold nanoparticles/carboxylated multiwalled carbon nanotubes@thionine connecting the complementary strand of the aptamer (AuNPs/cMWCNTs/cDNA@thionine), combined with the portable TFGE, can be used for the field detection of oxytetracycline. The reduction of AuNPs on the surface of the cMWCNTs yielded AuNPs-cMWCNTs, which have many binding sites, faster electron transfer rates, and are ideal biosensor materials.

**Figure 2:**
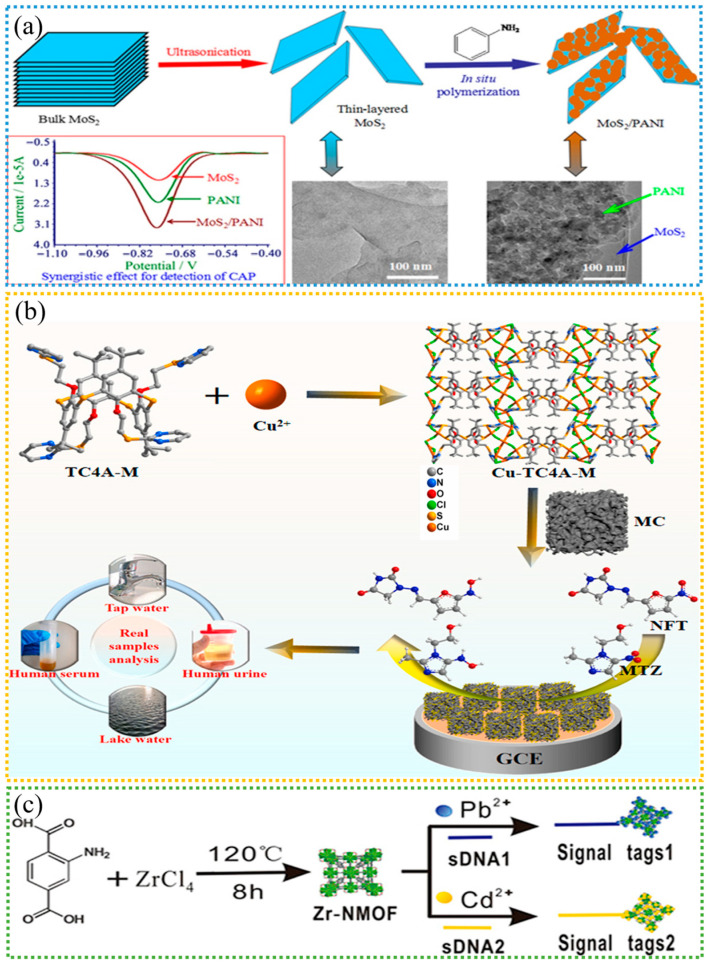
(**a**) The MoS_2_/PANI nanocomposite was synthesized via in situ polymerization of aniline in the presence of thin-layered MoS_2_, resulting in a synergistic effect for the detection of CAP due to the special structure and the physisorption interaction between aromatic aniline and MoS_2_. Reproduced with permission from Ref. [85]. Copyright 2016, Elsevier. (**b**) New Cu-TC4A-M@MC/GCE sensors were successfully prepared by combining a thiacalix[4]arene-copper (I)-based MOF with mesoporous carbon, which featured efficient detection of nitrofurantoin and metronidazole. Reproduced with permission from Ref. [88]. Copyright 2024, Elsevier. (**c**) Synthesis of UiO-66-NH_2_ nanoparticles and preparation of two signal tags. Reproduced with permission from Ref. [89]. Copyright 2017, Elsevier.

Further study of metal nanoparticles revealed that metal compound nanoparticles also have electrochemical properties similar to those of metal nanoparticles and are easier to chemically modify to achieve better electrocatalytic effects, thus improving the sensitivity of the sensor. Owing to their superior chemical and physical properties, transition metal oxides have been widely used in electrochemical sensing. Chen et al. prepared bulk MoS_2_ by ultrasonic exfoliation and obtained a molybdenum disulfide/polyaniline (MoS_2_/PANI) nanocomposite with a three-dimensional special structure via in situ polymerization of aniline [85]. MoS_2_ is a layered transition metal sulfide composed of three atomic layers stacked by weak van der Waals forces, exhibiting a structure similar to graphene. When integrated with other functional materials, MoS_2_ can enhance performance due to synergistic effects. As shown in Figure 2a, MoS_2_ has a relatively smooth layered structure, and PANI nanoparticles appear on the surface and interlayers of the layered MoS_2_ to form a 3D structure. Both MoS_2_ and PANI catalyze the electrochemical reduction of chloramphenicol (CAP). However, the catalytic effect of MoS_2_ is relatively poor, which may be attributed to its low electrical conductivity. Nevertheless, the MoS_2_/PANI composite results in a greater reduction peak current, indicating a synergistic catalytic effect of MoS_2_/PANI on CAP. This may be attributed to the physical adsorption interactions between aromatic PANI and the MoS_2_ substrate, as well as the excellent electrochemical performance of the MoS_2_/PANI nanocomposite. Additionally, the unique structure of the MoS_2_/PANI nanocomposite enables efficient adsorption of conjugated CAP, thereby promoting electron exchange between CAP and the electrode surface and providing an excellent platform for CAP detection. In addition, Rajaji et al. prepared tiny hierarchical manganese(III) oxide nanostructures (Mn_2_O_3_ TNS) via a sonochemical method [35]. The prepared Mn_2_O_3_ TNSs are in the form of tiny flower-like architectures and contain nanoporous structures. When Mn_2_O_3_ TNS was used to modify the electrode, the catalyst promoted sufficient active sites to attract more reactants, resulting in higher reduction peak currents and minimal overpotentials. Moreover, owing to the good stability and high activity of Co_3_O_4_, Yadav used a hydrothermal method to generate Co_3_O_4_ nanocrystals on the surface of reduced graphene oxide (Co_3_O_4_@rGO) for use as electrochemical sensing materials [86]. For the quantitative detection of chloramphenicol, the sensor GC/Co_3_O_4_@rGO reduces the nitro group of chloramphenicol to hydroxylamine, producing a sensitive irreversible reduction peak. In addition, metal ions can present different valence states, and oxidation or reduction reactions will occur during the electrochemical reaction, thus generating electrochemical signals. Therefore, various metal ions can be utilized as electrochemical signal probes for the indirect detection of target antibiotics [87].

Metal–organic frameworks (MOFs) are porous materials with periodic network structures formed by self-assembly of metal ions or metal clusters with organic ligands through coordination bonds [90]. MOFs have extremely high specific surface areas and rich pore structures, and their pore size and properties can be precisely regulated by selecting different metal ions and organic ligands. In addition, MOFs can maintain the structure and performance stability under different conditions. Due to their highly controllable structures, mechanical strength, sustainability and environmental friendliness, MOFs and their derivative composites have been commonly used in materials for flexible electrochemical sensors in recent years [91]. MOFs can also be modified by ions or combined with other materials to achieve different properties [89]. Ma et al. composited MOFs with carbon materials to avoid the accumulation of MOFs and improve the electrical conductivity [88]. As shown in Figure 2b, thiacalix[4]arene-copper(I) metal-organic framework (Cu-TC4A-M@MC), a nanocomposite material of Cu-based MOF (Cu-TC4A-M) and mesoporous carbon (MC), is an electrochemical sensor modification material that can accurately detect antibiotics such as furantoin and metronidazole in real samples. Neighboring Cu(I) ions were linked together through the Cu(I)–Cl bonds and Cu(I)–Cu(I) interactions to produce a [Cu_9_Cl_9_]_n_ chain. Two adjacent chains are further connected via TC4A-M ligands through Cu(I)–S and Cu(I)–N bonds, forming a stable two-dimensional network. Additionally, the catalytic performance of the composite material (Cu-TC4A-M@MC) is superior to that of Cu-TC4A-M and MC, indicating that synergistic catalysis plays a role in the reaction process. First, NFT is adsorbed by Cu-TC4A-M, and NFT is reduced to Red-NFT by obtaining electrons from MC. Then, Red-NFT is transferred to the MC surface, and finally, Red-NFT diffuses into the solution. In addition, porous nanoscale MOFs with high loading capacities for metal ions are suitable for signal development in aptamer sensors. Chen et al. chose metal ion-encoded nanoscale metal–organic frameworks (NMOFs) as substrates for aptamer sensors to generate distinguishable signals [89]. As shown in Figure 2c, sDNA was encapsulated on M-NMOF by an amine-glutaraldehyde reaction to form a signal tag. In addition, the introduction of -NH_2_ of UiO-66-NH_2_ provides additional adsorption sites in the framework structure, thereby increasing the loading of Pb^2+^ and Cd^2+^. In the presence of the targets, the signal tags were released; thus the electrochemical signal was amplified. However, electrochemical sensors made from unmodified MOFs face two major challenges: limited conductivity and suboptimal stability. Owing to the limited overlap between d orbitals and p orbitals in the metal center, MOFs typically exhibit poor conductivity [92]. In addition, many of the bound organic ligands have inherent insulating properties. Therefore, most MOFs need to be modified before they can be used as electrode surface coatings.

### 3.3. Molecular Imprinted Polymers

Molecular imprinting technology (MIT) aims to construct a polymer with the ability to specifically recognize the template molecule. MIT typically involves template molecules, functional monomers, crosslinking reagents, etc. Functional monomers and templates form complexes through non-covalent (hydrogen bonds, ions or hydrophobic) and covalent interactions, and then undergo cross-linking reactions in solvents [93]. After the template plate was removed, molecularly imprinted polymers (MIPs) with three-dimensional microcavities were generated, complementing the template in shape and chemical function [94].

The combination of MIPs and electrochemical detection techniques makes detection sensitive, simple, and fast, enabling real-time detection in the field. However, MIP materials often face the problem of low electrical conductivity, which makes it difficult for the detection sensitivity to meet the actual demand. The introduction of nanomaterials has become an effective strategy to increase electrical conductivity. For example, by oscillating and incubating a molecularly imprinted polymer (MIP) of furacillin (NFZ) at low temperatures, Cheng used the fabricated MIP to construct a highly sensitive electrochemical detector that was specifically recognized by hydrogen bonds [95]. Owing to the lack of electrical conductivity of MIPs, nanocomposites of MOF and biochar (BC) have been introduced to improve the electrical conductivity. This sensor has good selectivity for the quantitative analysis of NFZ and provides a promising way to monitor NFZ in biological fluids.

Although MIP-based electrochemical sensors have the advantages of high selectivity and low fabrication cost, deep embedding and incomplete elution occur in the preparation of imprinted polymers in practical applications. Surface molecularly imprinted polymers (SMIPs) have advantages in minimizing the template-embedding problem in the template removal step [96]. SMIP involves the formation of molecular imprinting on the surface of solid substrates, which not only solves the problem of incomplete elution of template molecules but also facilitates faster identification and binding of analytes during the detection process. In electrochemical detection, electrochemical polymerization is often used to prepare molecularly imprinted polymers to form a certain thickness and uniform film [97]. A powerful electrochemical sensor with specificity was constructed from a Au nanoparticle-functionalized black phosphorus nanosheet nanocomposite (BPNS-AuNP) coated with a polypyrrole-imprinted film [98]. The BPNS-AuNP possessed high stability and electrocatalytic activity, which provided support for the specific recognition of the MIP. The MIP/BPNS-AuNP/GCE sensor enables ultrasensitive detection of nanomolar levels of norfloxacin. However, the binding mechanism of template molecules remains unclear, and MIPs have certain shortcomings in terms of stability and affinity for antibiotics. When the sample matrix is complex, pretreatment is required before detection via MIP electrochemical sensors, which prevents rapid and direct identification [40].

### 3.4. Covalent Organic Frameworks

Covalent organic framework (COF) materials are a new type of organic porous crystal polymer formed by light elements (B, C, Si, N, and O) through a number of precise atomic space combinations, discrete voids, and strictly ordered covalent bonds (B-O, C-N, C=N, C=C-N) of organic architecture [99]. COFs are widely used in various fields because of their unique properties, such as ordered pore structure, large specific surface area, highly adjustable porosity, selectable construction units, predictable structure and abundant functional groups [100,101]. According to the different construction dimensions, COFs can be divided into 2D COFs and 3D COFs. 2D COFs are a class of two-dimensional crystalline porous materials, usually composed of planar aromatic monomers, that exhibit π electron delocalization in both the parallel and vertical directions, facilitating charge transport. However, 2D COF layers are often densely packed, resulting in the formation of one-way one-dimensional channels where many active sites are buried, limiting their electrical properties. Compared with 2D COFs, 3D COFs have a more abundant pore structure, usually have a greater specific surface area, and can provide more active sites. However, the synthesis of 3D COFs is difficult and still faces many challenges [102].

Although COF materials have high porosity and good stability, their electrical conductivity is relatively poor, especially in terms of electron transport. To improve the conductivity of COF materials, they are often combined with other materials to achieve better detection performance [103,104]. Sun et al. developed a novel 3D electrochemical sensor, MIP/MoS2/NH2-MWCNT@COF/GCE, by combining nanomaterial-modified COFs with MIP technology [60]. Owing to its high electrical conductivity, regular pore channels and excellent crystallinity, NH2-MWCNT@COF greatly enhanced the electrochemical performance. NH2-MWCNT@COF with MoS2 nanosheets formed a 3D structure on the electrode surface and enabled dual signal amplification. The electrode surface was further modified by electrochemical polymerization with MIP, which served as a probe for the selective recognition of sulfamethazine. Au NP-embedded covalent organic frameworks (Au@COFs) were prepared via the impregnation–reduction method [105]. The interactions between the Au NPs and O atoms in the COFs were evenly distributed throughout the COF. Aptamers can strand on the COF surface via strong π–π stacking, and the Au NPs increase the electrical conductivity. This strategy provides a feasible way to develop electrochemical detection sensors with metal nanoparticle-built-in COF composites. Compared with MOFs, COFs exhibit superior thermal and chemical stability due to their covalent bonds [106]. However, there are currently few studies on the use of pure COFs for electrochemical detection, with most research focusing on composite materials incorporating other components. This field remains in its infancy and requires further development.

### 3.5. Biological Materials

Electrochemical biosensors use biomolecules or biomimetic reagents to specifically recognize the target analyte and convert biological cognitive components into electrochemical signals [107,108]. The unique structure of nanomaterials can provide a location for bioactive ingredients to attach, which is conducive to their immobilization and use, thus improving their detection efficiency. Biological materials, such as enzymes, proteins and cells, have specific recognition capabilities and can generate signals through specific reactions with target molecules to achieve qualitative or quantitative detection. The molecular recognition elements in biosensors can be divided into several categories: electrochemical protein sensors, electrochemical immunosensors, electrochemical aptamer sensors, and electrochemical microbial sensors.

**Figure 3:**
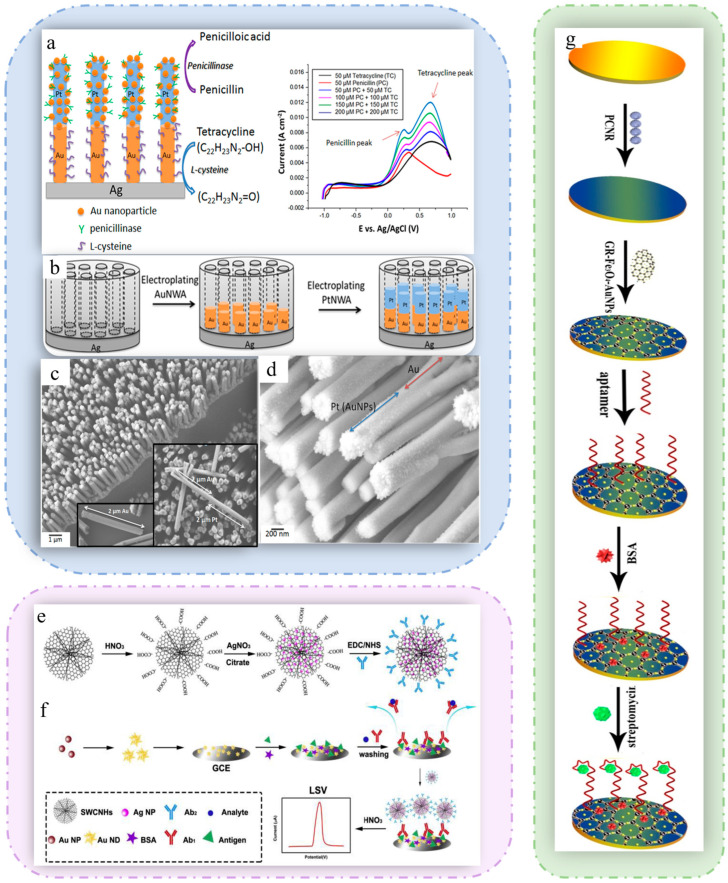
(**a**) Fabrication process of the Au-Pt multisegment nanowire array. FESEM images of (**b**) a Au-Pt multisegment nanowire array, where the insets are a single Au nanowire and a single Au-Pt nanowire; (**c**) AuNPs coated on a Pt segment after being treated with 3 mM HAuCl4 solution; (**d**) penicillin and tetracycline. (**a**–**d**) Reproduced with permission from Ref. [109]. Copyright 2019, Elsevier. (**e**) Synthesis steps of Ag NPs@SWCNHs@Ab2. (**f**) Schematic illustration of the preparation of Ag NPs@SWCNHs@Ab2/Ab1/Cag/Au ND@GCE and the mechanism of the competitive immune system for detecting SMZ. (**e**,**f**) Reproduced with permission from Ref. [110]. Copyright 2019, Elsevier. (**g**) Schematic diagram of the streptomycin aptasensor. Reproduced with permission from Ref. [111]. Copyright 2017, Elsevier.

The electrochemical protein sensor uses protein as a sensitive element to generate electrical signals through the reaction of the protein with the target object. Antibiotic enzymes are important bioactive components for detecting antibiotics; they can bind to antibiotics specifically and chemically react with them. An electrochemical biosensor with a new hybrid array was successfully developed [109] and can be used to detect penicillin and tetracycline simultaneously, as shown in Figure 3a. As shown in Figure 3b, a Au-Pt multisegment nanowire array was fabricated by electrodeposition within anodized aluminum oxide (AAO) films, and the length of the Au or Pt segments could be controlled by changing the conditions of deposition. Figure 3c shows the morphology of the Au-Pt multisegment nanowire array, which is illustrated with a single Au nanowire and a single Au-Pt nanowire. A monolayer of L-cysteine is fixed to the Au segment and acts as a bioreceptor for tetracycline detection. Furthermore, the Au NPs were uniformly loaded on the Pt segment via an electroless deposition procedure to immobilize penicillinase, and their morphology is shown in Figure 3d. In the multisegment nanowires/nanoparticles hybrid array structure, all nanowires are uniformly distributed and vertically aligned on a metal substrate, and there is no need to use Nafion to cover the functionalized nanowires on the glassy carbon electrode surface, resulting in lower signal noise and better sensor durability. The vertically aligned structure also increases the contact area between the nanowires and the analyte, resulting in higher sensitivity and lower detection limits.

Electrochemical immunosensors are usually based on antigen–antibody interactions [112], where the target antibiotic (antigen) binds specifically to the antibody fixed on the electrode surface to form an immune complex. This combination will cause changes in the electrochemical properties of the electrode surface, generate electrical signals, and then achieve quantitative detection of the target substance. Zhang et al. constructed an immunosensor that utilizes the competitive release of Ag^+^ and thus enhances electrochemical signals [110]. The acidified single-walled carbon nanohorns (SWCNHs) were enriched with -COOH to increase their water solubility, as shown in Figure 3e. Ag NPs were uniformly attached to the surface of the SWCNHs, and the outer layer was encapsulated by Ab2 to form Ag NPs@SWCNHs@Ab2. The Ab1/Cag/BSA/Au NDs/GCE was formed by drop-coating, in which Au nanodendrites (Au NDs) contributed to the enhanced electrical conductivity, as demonstrated in Figure 3f. In the presence of HNO_3_, Ag^+^ was released during the competition between the coating antigen (Cag)-Ab1 and Ag NPs@SWCNHs@Ab2, causing changes in the electrical signal.

An aptamer is a single-strand DNA or RNA sequence that is screened by exponential enrichment ligand phylogenetic evolution (SELEX) and can bind specifically to a variety of target molecules, including small molecules, proteins, and cells [113,114]. An electrochemical aptamer sensor for highly specific and sensitive detection of streptomycin was constructed by Yin [111]. As shown in Figure 3g, the sensor uses highly electrocatalytically active porous carbon nanorods (PCNs) and multifunctional graphene nanocomposites (GR-Fe_3_O_4_-AuNPs) as biosensing substrates. The aptamers were fixed on the electrodes through stable Au–SH covalent bonds. To ensure the specificity of the sensor, bovine serum albumin (BSA) was added to block nonspecific binding sites. The sensor, characterized by good reproducibility, high selectivity and stability, has been successfully used for the determination of streptomycin in milk.

### 3.6. Quantum Dots

Quantum dots (QDs), semiconductor nanocrystals with a size of nanometers, have good performance in the field of electrochemical detection because of their unique physical and chemical properties [115]. Compared with the conventional nanomaterials, QDs have emerged as promising alternatives due to their unique chemical and optical structural properties. QDs offer advantages such as excellent biocompatibility, ultra-small size, distinctive physicochemical properties, and low cost [116]. Quantum confinement effects and surface modifications are key points that attract researchers to build QD-based sensors, including electrochemical sensors. The electrochemical and photophysical properties of quantum dots play a leading role in electrochemical analysis [117]. In addition, quantum dots have size-dependent fluorescence characteristics, enabling fluorescence and electrochemical dual-mode detection. After surface modification, many quantum dots have good biocompatibility and can bind specifically to biomolecules without significantly affecting their activity [117].

Santos et al. synthesized CdTe quantum dots and prepared CdTe-CB-CTS:EPH/GCE for the detection of norfloxacin by modifying a glassy carbon electrode with carbon black and CdTe quantum dots in a chitosan membrane [66]. Chitosan (CTS)/epichlorohydrin (EPH) forms an adherent, homogeneous film on a GCE with high electrical conductivity. The acceptance groups of CTS:EPH interacted with the -COOH groups on the surface of the CB and CdTe particles to immobilize the CB and CdTe nanomaterials at the electrodes. The action of CB and CdTe QDs on the surface of the electrodes enhanced the electrochemical signals. In addition, the sensor exhibited good electrocatalytic behavior for the oxidation of norfloxacin in aqueous media, which enabled cost-effective, sensitive and rapid detection. Li et al. developed a sensitive dual-labeled aptasensor using CdS and PbS QDs for the simultaneous detection of multiple antibiotics [118]. In the presence of the target antibiotics, the CdS and PbS quantum dots were released and dissolved into the metal ions. The electrochemical peak signals were enhanced due to the presence of many metal ion markers. Different metal ion-labeled aptamers respond differently to different analytes, thus enabling the simultaneous detection of antibiotics. Although promising, QDs easily aggregate and are difficult to synthesize, which hinders their biosensing applications [119]. Therefore, the application of quantum dots in electrochemical detection is still challenging.

## 4. Application

To adapt to the rapid real-time monitoring of antibiotics in different scenarios, portable electrochemical detection platforms appeal to the public. Combining screen printing, chip, smartphone or artificial intelligence technologies with electrochemistry to build portable detection platforms can overcome site restrictions and yield real-time detection results at home, in the field and at other sites, and the operation is simple and user friendly.

As a type of portable device, a wearable electrochemical detection platform is a smart device that integrates miniaturized sensors and signal processing circuits. It utilizes electrochemical principles and can quickly and accurately detect various chemical substances through contact with the surface of skin or food. For example, for human health detection, it can monitor metal ions, biomarkers and drugs [120,121,122,123] in sweat in real time, and these indicators can help manage drug use and assess potential disease risk. Portable electrochemical detection platforms are also usually combined with smartphones [124,125], providing convenient, efficient and real-time results. Electrical signals can be transmitted to the smartphone via Bluetooth, Wi-Fi or type-C ports, which further analyze and store the signals. These electrical signals are presented to the user in the form of intuitive charts, numbers, etc., enabling real-time monitoring and rapid detection.

Here, portable electrochemical sensors will be introduced from four aspects: clinical diagnostics, food safety, environmental monitoring and pharmaceutical analysis.

### 4.1. Clinical Diagnostics

**Figure 4:**
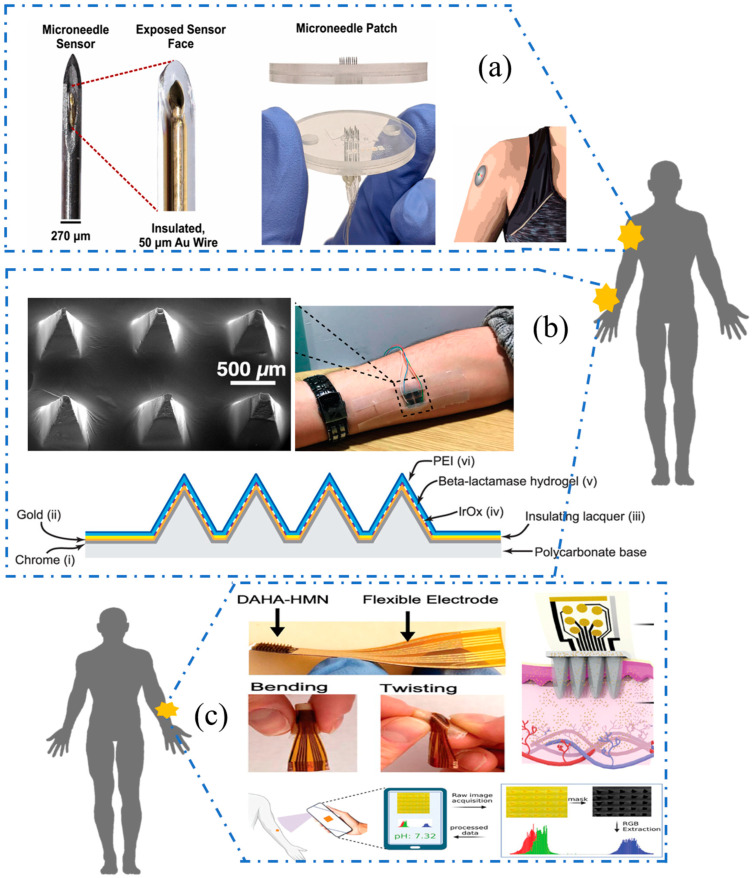
(**a**) Details of wearable transdermal EAB sensing. A 3 × 3 array of needles (containing sensors and a single reference and counter electrode) was placed into a laser-cut polymethylmethacrylate (PMMA) housing. This allows penetration of the skin in a wearable format. Reproduced with permission from Ref. [126]. Copyright 2023, Elsevier. (**b**) Schematic cross-section of working electrode microneedles showing each layer and SEM image of a microneedle array coated with iridium oxide and enzyme hydrogel layers. Reproduced with permission from Ref. [127]. Copyright 2019, American Chemical Society. (**c**) Schematic illustration of the HMN-Flex assay, which represents the bent and twisted states, working mechanism and automated process of cropping, masking, RGB extraction, and pH evaluation. Reproduced with permission from Ref. [128]. Copyright 2024, Wiley.

In clinical practice, microneedle sensors worn on the epidermis can assist in monitoring antibiotic content in the human body. Downs et al. assembled a wearable electrochemical aptamer-based (EAB) sensor by embedding a gold microfilament sensor into a stainless steel insulin microneedle and immobilizing the microneedle sensor array into a polymethylmethacrylate (PMMA) housing. As shown in Figure 4a, 3 × 3 needle arrays with adjustable needle lengths enabled penetration of the skin in a wearable form. This EAB sensor detected vancomycin in undiluted blood at body temperature and was capable of clinical monitoring [126]. It is not uncommon for microneedle-based sensors to be used to detect antibiotics. As shown in Figure 4b, Gowers et al. developed a minimally invasive biosensor based on minimally invasive microneedles that only penetrate the cuticle of the skin and do not cause pain or draw blood. The sensor was able to track penicillin concentrations in volunteers in real time [127]. Keyvani et al. developed a biosensor consisting of hydrogel microneedles (HMNs) and an aptamer-functionalized flexible electrode (Flex) for monitoring vancomycin and gentamicin levels. After HMNs are extracted from the dermal interstitial fluid, they are transferred to the Flex electrode, where the target antibiotic can be detected. As shown in Figure 4c, HMN-Flex can still maintain good performance in the state of bending or twisting, meeting the requirements of wearing [128]. The performance of some wearable electrochemical sensors is already comparable to commercial devices [129,130,131]. Thus, it seems that the development of wearable antibiotic sensors also has great potential for commercialization and practicality.

### 4.2. Food Safety

**Figure 5:**
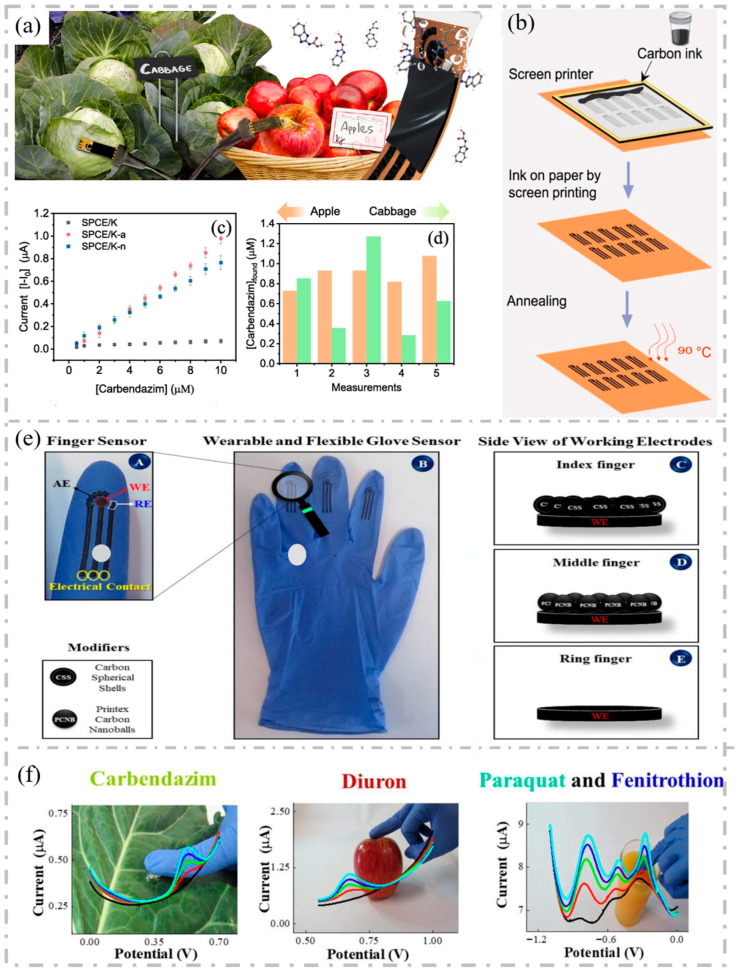
(**a**) Setup used for detecting carbendazim on apple and cabbage. (**b**) Illustration of the preparation process of screen-printed carbon electrodes. (**c**) Analytical curves for carbendazim on SPCE/K-n, SPCE/K-a, and SPCE/K. (**d**) Found concentrations of carbendazim on the surface of apple and cabbage with the SPCE/K-a sensor. Reproduced with permission. (**a**–**d**) Reproduced with permission from Ref. [132]. Copyright 2023, Elsevier. (**e**) Details of the finger sensor design with a complete electrochemical system. A: Details of finger sensor design with a complete electrochemical system: auxiliary, reference and working electrodes. The connection between electrodes and potentiostat was made via flexible conductive wires for on-site detection. B: Image of the real screen-printed sensing glove. C, D, E: Schematic representation of the side views of CSS, PCNB and pretreated sensing layers for index, middle and ring fingers, respectively. (**f**) Photos of actual measurement conditions with wearable glove-embedded sensors on food samples of cabbage, apple and orange juice. Each photo is accompanied with the corresponding electrochemical screening of SWV signature (the black one) and the voltammograms using four different multidimensional projection techniques (the other four colors). (**e**,**f**) Reproduced with permission from Ref. [133]. Copyright 2021, Elsevier.

In food safety testing, antibiotic residue levels in food are detected by wearing glove-like wearable sensors. As shown in Figure 5a–d, Martins et al. made screen-printed electrodes on kraft and parchment papers with conductive carbon ink to construct wearable sensors suitable for acid or neutral solutions to detect carbendazim on the surface of apples and cabbage without destruction [132]. Raymundo-Pereira et al. embedded three nonenzyme sensors on three fingers of rubber gloves, as shown in Figure 5e,f, to detect residues of pesticides on the surface of food and directly detected analytes in combination with functionalized sensing layers and electrochemical pretreatment [133]. Here, square wave voltammetry (SWV) was applied to detect different analytes. The high performance and discrimination of the analytes at distinct concentrations in real samples of apple and cabbage by simply touching with the glove, and in orange juice by immersing the fingers was demonstrated with four multidimensional projections. The sensor offers the possibility of detecting more chemicals, including antibiotics.

### 4.3. Environmental Monitoring

Residual antibiotics can also have an impact on the environment. Monitoring of antibiotics in the environment is often carried out using portable devices such as screen-printed electrodes (SPE) or smartphones.

**Figure 6:**
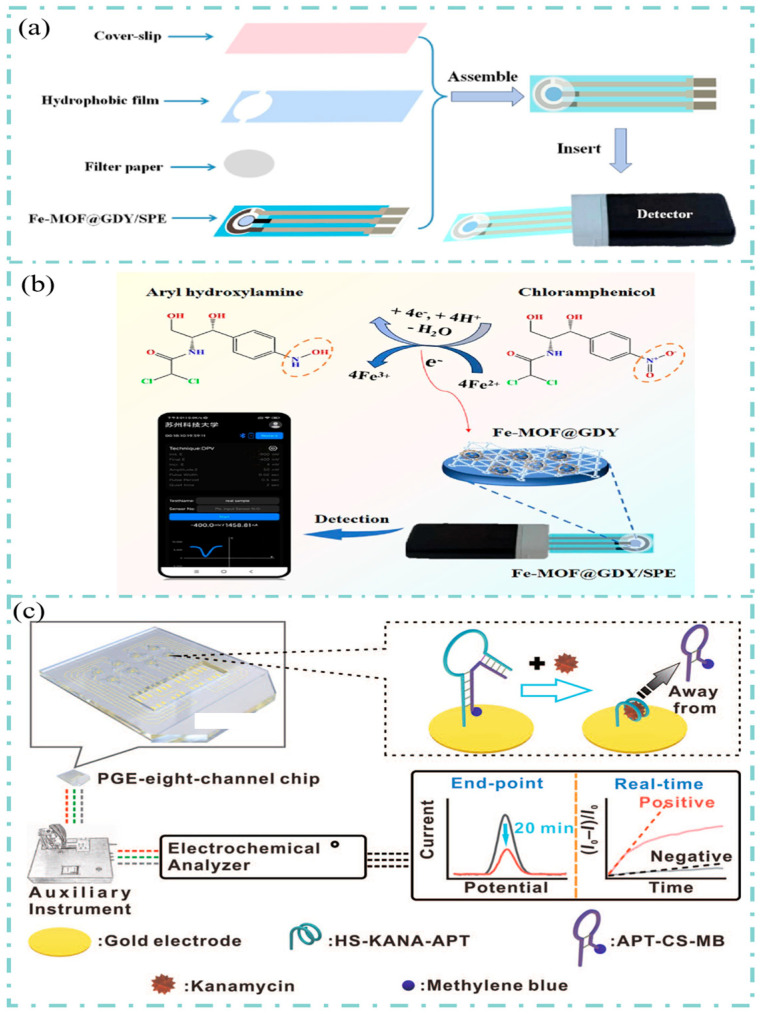
(**a**) Configuration of the portable Fe-MOF@GDY-based chip device. (**b**) Schematic illustration of Fe-MOF@GDY and its electrochemical sensing chip for on-site portable and wireless detection of antibiotic CAP. Reproduced with permission from Ref. [134] Copyright 2024, Elsevier. (**c**) Schematic diagram of kanamycin electrochemical detection on the PGE-eight-channel chip and device combination. Reproduced with permission from Ref. [135] Copyright 2023, Wiley.

Zhang et al. prepared a portable electrochemical chip that can be combined with a smartphone to monitor chloramphenicol in lake water samples [134]. As shown in Figure 6a, the portable electrochemical chip consists of four integrated layers: a protective film, a hydrophobic film, a filter membrane, and an electrode. The principle of the portable device monitoring of chloramphenicol was shown in Figure 6b. On the SPE electrode, Fe-MOF acts as the active site of the nanocomposite, interacting with chloramphenicol to generate an electrical signal. The portable electrochemical detection module, which is compatible with SPE, transmits the electrochemical signal to a smartphone via Bluetooth.

Bao et al. developed a portable electrochemical device capable of rapid and real-time detection of kanamycin residues in actual water samples [135], as shown in Figure 6c. Gold electrodes on a polystyrene substrate (PGE) prepared by chemical vapor deposition. The kanamycin aptamer ssDNA (HS-KANA-APT) is modified on the PGE surface via Au-S covalent bonds and forms a conjugated structure in the presence of complementary DNA chains (named APT-CS-MB in Figure 6c). When kanamycin is present, it can form a more stable and complex structure with HS-KANA-APT. Meanwhile, the weakly bound APT-CS-MB is competitively displaced from the electrode surface, leading to differences in the electrical signal.

### 4.4. Pharmaceutical Analysis

Portable electrochemical sensors, with their miniaturization, rapid response and high sensitivity characteristics, have been effectively applied in the field of pharmaceutical analysis.

**Figure 7:**
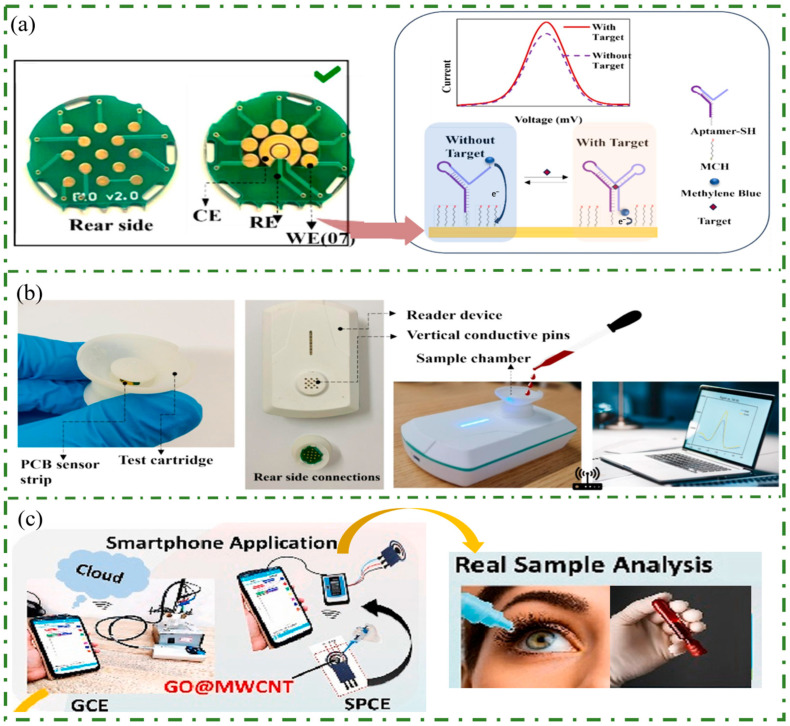
(**a**) The diagram of the designed PCB electrode chip and the detection principle diagram. (**b**) The integrated diagram of the complete sensor platform. Reproduced with permission [136]. Copyright 2024, Elsevier. (**c**) A smartphone-assisted platform for CAP detection, enabling wireless, Bluetooth-enabled, and real-time analysis via a user-friendly smartphone application performed effectively in eye drops, capsules, and human blood serum. Reproduced with permission [137]. Copyright 2025, Elsevier.

Liu et al. developed a portable biosensor based on electrochemical aptamers, which has broad application potential in pharmaceutical analysis and is commercially feasible [136]. They designed a novel disc-shaped test chip with contact points on its rear side, which established reliable connections with electrodes via printed circuit board (PCB) vias. The sensing principle diagram of the working electrode on the chip is shown in Figure 7a. Changes of electron transfer kinetics caused by binding to the target result in current changes. Additionally, a wireless and compact signal processing module (named ZiOstat) has been integrated. On one hand, ZiOstat can convert the highly sensitive current response into a transduction signal. On the other hand, a wireless connection has been established between ZiOstat and the user interface to ensure smooth experimental operation and data acquisition. The complete sensor platform, shown in Figure 7b, includes ZiOstat, a test box capable of mounting a functionalized PCB electrode chip, and an external laptop interface.

Dogra et al. used a Screen-Printed Carbon Electrode (SPCE) combined with a smartphone to perform quantitative analysis of chloramphenicol (CAP) in pharmaceutical eye drops, capsules and serum [137], as shown in Figure 7c. To facilitate on-site detection of CAP, the electrochemical setup was miniaturized by using a PalmSens pocket potentiostat (a portable electrochemical device made by PalmSens B.V., The Netherlands) and integrated it with a smartphone via Bluetooth using the PS Touch Android software. Additionally, to enhance sensing capabilities, the SPCE was modified with GO@MWCNT nanocomposites. Compared to traditional laboratory electrochemistry instruments, the smartphone-assisted platform demonstrated good consistency, validating its reliability and analytical performance.

## 5. Conclusions and Prospects

This review describes the progress in the use of electrochemical sensors in the detection of antibiotics in terms of analytical methods, materials and applications. As the core component of sensors, which is also the focus of this review, modified materials are classified according to their properties. Carbon and metal nanomaterials usually have high electronic conductivities. MOFs are porous and easy to combine with other materials. Like MOFs, COFs have high porosity and good stability. MIPs can achieve specific selection on the basis of their imprinted holes matching with target molecules. Biomaterials have the ability to specifically identify and target molecules. In fact, composite materials can combine the advantages of multiple materials and are more commonly used in practical testing. Additionally, the applications of portable electrochemical sensors in four different aspects are introduced.

Although electrochemical sensors have some advantages, they still face some challenges and need improvement. At present, electrochemical sensors for detecting antibiotics still have limitations. In addition, the amount of antibiotic residue in the environment is usually at the nM level or lower, so the sensitivity of electrochemical detection also needs to be improved. Simplifying the pretreatment steps of samples, shortening the analysis time, increasing robustness and establishing a rapid, simple and low-cost analysis method that can simultaneously detect multiple antibiotic residues are urgent problems to be solved. Concurrently, the miniaturization of instruments to achieve high sensitivity and real-time monitoring in the field has become a development trend, in which wearable devices have great potential.

## Figures and Tables

**Table 1:** Overview of antibiotics.

Antibiotics Class	Example	Target Microbes	Mechanism of Action	Side-Effects	Origin
Macrolides	Erythromycin, roxithromycin, clarithromycin	G^+^, G^−^	Inhibit protein synthesis	Vomiting, diarrhea, rash, fever	Microbial metabolism
Tetracyclines	Tetracycline, oxytetracycline, chlortetracycline	G^+^, G^−^	Inhibit protein synthesis	Nausea, abdominal pain, loss of appetite, vitamin deficiency	Microbial metabolism, artificial semi-synthesis
Bata-lactams	Penicillin, cephalosporin, cephalin	G^+^, G^−^, haemophilus	Destroy cell wall	Allergy	Microbial metabolism, artificial semi-synthesis
Sulfonamides	Sulfonamide, sulfadiazine, sulfonthiazole	G^+^, G^−^, nocardia, chlamydia	Inhibit folic acid synthesis	Allergy, kidney damage	Artificial synthesis
Aminoglycosides	Streptomycin, gentamicin, kanamycin	G^−^	Inhibit protein synthesis, destroy cell membrane	Nephrotoxicity, ototoxicity	Microbial metabolism, artificial semi-synthesis
Lincosamides	Lincomycin, clindamycin	G^+^, anaerobic bacteria	Inhibit protein synthesis	abdominal pain, diarrhea, allergy	Artificial semi-synthesis

G^+^: Gram-positive bacteria, G^−^: Gram-negative bacteria.

## Data Availability

Not applicable.
